# Astaxanthin Extract from *Haematococcus pluvialis* and Its Fractions of Astaxanthin Mono- and Diesters Obtained by CCC Show Differential Antioxidant and Cytoprotective Effects on Naïve-Mouse Spleen Cells

**DOI:** 10.3390/antiox12061144

**Published:** 2023-05-24

**Authors:** Zuzana Jurčacková, Denisa Ciglanová, Dagmar Mudroňová, Lenka Tumová, Daniela Bárcenas-Pérez, Jiří Kopecký, Jana Koščová, José Cheel, Gabriela Hrčková

**Affiliations:** 1 Institute of Parasitology, The Slovak Academy of Sciences, Hlinkova 3, 04001 Košice, Slovakia; jurcackova@saske.sk (Z.J.); ciglanova@saske.sk (D.C.); 2 Department of Microbiology and Immunology, University of Veterinary Medicine and Pharmacy, Komenského 68, 04181 Košice, Slovakia; dagmar.mudronova@uvlf.sk; 3 Department of Pharmacognosy and Botany, Faculty of Pharmacy Hradec Králové, Charles University, Heyrovského 1203, 50165 Hradec Králové, Czech Republic; tumova@faf.cuni.cz; 4 Laboratory of Algal Biotechnology—Centre ALGATECH, Institute of Microbiology of the Czech Academy of Sciences, Opatovický mlýn, 37981 Třeboň, Czech Republic; barcenas@alga.cz (D.B.-P.); kopecky@alga.cz (J.K.); jana.koscova@uvlf.sk (J.K.); 5 Faculty of Science, University of South Bohemia, Branišovská 1760, 37005 České Budějovice, Czech Republic

**Keywords:** astaxanthin, monoesters, diesters, *Haematococcus pluvialis*, mouse, splenocytes, oxidative stress, viability, countercurrent chromatography (CCC)

## Abstract

Carotenoids are the most abundant lipid-soluble phytochemicals and are used as dietary supplements to protect against diseases caused by oxidative stress. Astaxanthin, a xanthophyll carotenoid, is a very potent antioxidant with numerous beneficial effects on cellular functions and signaling pathways. In this study, using spleen cells from healthy Balb/c mice, we report the bio-functional effects of an astaxanthin-rich extract (EXT) prepared from the microalga *Haematococcus pluvialis* and its astaxanthin monoesters-rich fraction (ME) and astaxanthin diesters-rich fraction (DE) obtained by fractionation of EXT using countercurrent chromatography (CCC). After incubation under standard culture conditions (humidity, 37 °C, 5% CO_2_, atmospheric oxygen), the viability of untreated splenocytes, as determined by the trypan blue exclusion assay, the MTT assay, and the neutral red assay, decreases to approximately 75% after 24 h compared with naïve splenocytes. This effect correlated with the decrease in mitochondrial membrane potential and the transition of ~59% of cells to the early stage of apoptosis, as well as with the decreased ROS production, indicating that hyperoxia in cell-culture deteriorates cell functions. They are restored or stimulated by co-cultivation with EXT, ME, and DE up to 10 µg/mL in the order EXT > DE > ME, suggesting that esterification increases bioavailability to cells in vitro. ROS and H_2_O_2_ concentrations reflect mRNA transcriptional activity of Nrf2, superoxide dismutase 1 (SOD1), catalase, and glutathione peroxidase 1, as well as SOD-mediated ROS conversion, whereas they inversely correlate with iNOS-mediated NO production. The highest-tested concentration of EXT, ME, and DE (40 µg/mL) is detrimental to cells, probably because of the overwhelming scavenging activity of astaxanthin and its esters for the reactive oxygen/nitrogen species required for cellular functions and signal transduction at low physiological concentrations. In this study, we demonstrate that differential activities of ME and DE contribute to the final antioxidant and cytoprotective effects of astaxanthin extract, which is beneficial in preventing a wide range of ROS-induced adverse effects, with DE being more effective. In addition, the selection of physioxia-like conditions for pharmacological research is highlighted.

## 1. Introduction

Reactive oxygen species (ROS) serve as important signaling molecules in physiological processes. However, an imbalance between the production of ROS and the antioxidant defenses that protect cells has been linked to the development of a number of diseases, including cancer, asthma, pulmonary hypertension, heart disease, autoimmune and metabolic diseases, and others. Great attention is also paid to the role of ROS overproduction in human aging [1,2]. In the cells, ROS are generated by numerous sources, including the mitochondrial respiratory chain as the main source, NADPH oxidases, nitric oxide synthases, lipoxygenases, and others [3]. Under physiological conditions, the ROS levels are regulated and maintained at low levels by antioxidant systems. ROS may be involved in many cellular metabolic pathways by modulating a number of kinases, phosphatases, redox-sensitive transcription factors, and genes that contribute to the regulation of cell growth, differentiation, proliferation, and apoptosis [4]. The overproduction of ROS can be endogenous and is mainly attributed to innate immune cells, macrophages, and neutrophils, as part of the defense against pathogens. However, the source of ROS may also be exogeneous, e.g., ultraviolet light, ionizing radiation, or drugs [5]. Higher nonphysiological levels of ROS in the form of superoxide, hydrogen peroxide, and other oxidants can be harmful to mammalian cells, leading to lipid peroxidation, DNA damage, and protein oxidation. Chronic oxidative stress can lead to alterations in signal transduction, mitogenesis, and cell death/apoptosis [3,4].

In the presence of persistent or chronic oxidative stress in the cells, the functions of endogenous antioxidant regulatory systems, particularly glutathione, the enzymes SOD, catalase, and glutathione peroxidase, are often alleviated or suppressed, and additional antioxidant sources from the diet are required [6]. In particular, these include vitamin E (tocopherol), vitamin C (ascorbate), polyphenolic antioxidants, and carotenoids. Among carotenoids, the great antioxidant capacity of astaxanthin has been highlighted and reported to be superior even to that of other known antioxidants [7,8]. Astaxanthin (AXT; 3,3′-dihydroxy-β, β′-carotene-4,4′-dione) belongs to the group of xanthophyll carotenoids [9]. In nature, astaxanthin is synthesized mainly by microorganisms—*Brevibacterium*, *Mycobacterium lacticola*, *Agrobacterium auratim*, microalgae—*Haematococcus pluvialis*, *Chlorella zofingiensis*, *Chlorococcum*, and yeast—*Phaffia rhodozyma*. On a commercial basis, astaxanthin is obtained from the microalgae *Haematococcus pluvialis* and the yeast *Xanthophyllomyces dendrorhous* (also called *Phaffia rhodozyma*) and by chemical synthesis. Currently, the most important natural source of astaxanthin is the microalga *Haematococcus pluvialis*, which is commonly used as a human dietary supplement [10]. Astaxanthin from *H. pluvialis* is commercially available mainly in the form of extract–oleoresin, obtained by the extraction with CO_2_ or with approved organic solvents [11,12]. From this oleoresin, only 10–15% accounts for the total astaxanthins. Other sources of astaxanthin such as synthetic astaxanthin and astaxanthin extracted from *Phaffia* yeast are used in animal nutrition and are not considered for direct human consumption [13]. Pharmacokinetic analysis of the organ distribution of synthetic astaxanthin in rats after oral application in food for two weeks showed that there was rapid uptake by the liver, but the highest concentrations of astaxanthin were found in the spleen, kidneys, and adrenals [14,15].

The effects of astaxanthin are based on its unique molecular structure, which consists of two carbonyl groups, two hydroxyl groups, and 11 conjugated ethylene double bonds (Supplementary Material). Due to hydroxyl (OH) and keto- (C = O) residues on each ionic ring, it can be esterified, and its structure also leads to antioxidant properties. Astaxanthin is a donor of ROS-reactive electrons that terminate the chain reaction and convert reactive molecules into more stable, harmless products [7]. Astaxanthin has also been shown to quench singlet oxygen, scavenge superoxide, hydrogen peroxides, and hydroxyl radicals, and inhibit lipid peroxidation [16]. Studies on the effects of astaxanthin have shown that it is a more potent antioxidant than β-carotene, inhibits lipid peroxidation more effectively than canthaxanthin, β-carotene, and zeaxanthin [7,8], and has no prooxidant effect, unlike lycopene, β-carotene, and lutein [17]. 

Nowadays, there is an increasing interest in this natural antioxidant compound because of its potential to alleviate age-related diseases. The astaxanthin-rich extract from *H. pluvialis* has been shown to protect the human body against a variety of diseases, including inflammation, gastric lesions, and cardiovascular disease, as well as liver disease, diabetes, cancer, UV-radiation-induced oxidative damage, and neurodegenerative diseases [18]. This astaxanthin complex is present in the red stage of the microalga *H. pluvialis* in the form of esters, of which 70% are astaxanthin monoesters and 25% are astaxanthin diesters. Approximately 5% of the total astaxanthins correspond to the non-esterified form [11,19]. The health-promoting properties and safety profile of astaxanthin in the free, non-esterified form have been extensively studied and recognized; however, astaxanthin monoesters and astaxanthin diesters are biologically poorly understood, and their contribution to the claimed beneficial effects has not been comparatively demonstrated. A few studies have addressed their effects on immune cells. Immune cells that play an important role in inflammation and pathogenesis, such as lymphocytes, macrophages, and dendritic cells, are very sensitive to oxidative stress. For example, [20] demonstrated that an excess of ROS differentially affects T-cell receptor signaling pathways and induces T lymphocyte hypo-responsiveness [21]. The spleen is the key organ of immune defense and consists of larger populations of T and B lymphocytes and a smaller population of macrophages and dendritic cells. Spleen cells represent the most sensitive primary cell lines suitable for studying the mechanisms by which natural substances can affect oxidative stress and related physiological processes. In the study of [22], the authors demonstrated that unesterified astaxanthin prevented partially oxidative stress in human blood lymphocytes induced by a fatty acid mixture. 

In our study, we investigated the effects of *H. pluvialis* extract corresponding to the total carotenoid astaxanthin-rich fraction and astaxanthin monoesters and diesters obtained by countercurrent chromatography (CCC) on selected physiological functions, antioxidant enzyme gene expression, and scavenging capacity for cell-producing ROS/NO in unstimulated spleen cells from healthy mice cultivated in vitro. 

## 2. Materials and Methods

### 2.1. Chemicals and Reagents

Splenocytes in each assay were grown in RPMI 1640 medium (Biochrom-Merck, Germany) without sodium bicarbonate, with phenol red, 2 mM of stable glutamine supplemented with 10% heat-inactivated bovine fetal serum (Biochrom, Berlin, Germany), 100 U/mL penicillin, 100 μg/mL streptomycin, 10 μg/mL gentamicin, and 2.5 μg/mL amphotericin B (all from Sigma-Aldrich, St. Louis, MO, USA). This medium was designated as a complete medium (CM). The other chemicals used in this study were 3-(4,5-dimethylthiazol-2-yl)-2,5-diphenyltetrazolium bromide (MTT, AppliChem, Darmstadt, Germany), trypan blue solution (Biochrom, Berlin, Germany), dimethyl sulfoxide (DMSO, Invitrogen, Waltham, MA, USA), and 2′,7′-dichlorodihydrofluorescein diacetate (H_2_DCFDA, Invitrogen, Waltham, MA, USA). The following chemicals were purchased from Sigma-Aldrich (St. Louis, MO, USA): 3-amino-7-dimethylamino-2-methylphenazine hydrochloride (Neutral Red), 2,2′-azo-bis-(2-methylpropionamidine dihydrochloride (AAPH) 1 M stock solution in distilled water, Hanks balanced sodium salt (HBSS) without phenol red, horseradish peroxidase (HRP), and 123-rhodamine (stock solution 1 mg/mL in ethanol). The Griess reagent was prepared from 5% orthophosphoric acid, 1% sulphanilamide, and 0,1% N-(1-Naphthyl) ethylenediamine dihydrochloride; the standard was NaNO_2_. Other chemicals used, NH_4_Cl, EDTA, and sodium azide, were used for cell biology applications. Acetone (HiPerSolv Chromanorm, Fontenay-sous-Bois, France) from VWR was used for microalgal biomass extraction. Acetonitrile with a purity of 99.9% (HiPerSolv Chromanorm, Leuven, Belgium) and *n*-heptane with a purity of 99% (HiPerSolv Chromanorm, Leuven, Belgium) from VWR were used for CCC separation. Methanol with a purity of 99.99% (HiPerSolv, Chromanorm, Fontenay-sous-Bois, France) from VWR was used for HPLC analysis of astaxanthins. Methanol and water for HPLC-HRMS analyses were purchased from Sigma-Aldrich (Germany). *H. pluvialis* biomass was purchased from Algamo, s. r. o. (Mostek, Czech Republic). Commercial standards of free astaxanthin, cantaxanthin, and lutein were obtained from Sigma Aldrich (Germany), Honeywell—Fluka (Seelze, Germany), and Extrasynthese (Lyon, France), respectively. 

### 2.2. Preparation of Microalgae Extract 

For the preparation of *H. pluvialis* extract, 60 g of microalgal biomass was extracted with acetone (600 mL). The extraction process was assisted by sonication with an ultrasonic bath (K6 Kraintek, s.r.o., Podhájska, Slovakia) with a frequency of 38 kHz and an intensity of 47.7707 W/cm at 25 °C and for 30 min. The resulting suspension was centrifuged to remove insoluble particles. The same procedure was repeated three times with the same biomass. The supernatant of the three extractions was combined and evaporated under reduced pressure at 30 °C, yielding 22.05 g of dry extract, which was used for CCC production of carotenoid fractions rich in monoesters and diesters of astaxanthin.

### 2.3. Fractionation of H. Pluvialis Extract by Countercurrent Chromatography (CCC) 

Fractionation of *H. pluvialis* extract was performed using a Quattro LabPrep CCC (AECS-QuikPrep Ltd. Cornwall, UK) with a 0.9 L column (PTFE bore = 3.2 mm). The rotation speed of the column was controlled by a speed controller installed in the CCC instrument chassis. The mobile liquid phase for the chromatographic process was delivered by a Q-Grad pump (LabAlliance, State College, PA, USA). The separation process was monitored using a sapphire UV-VIS spectrophotometer (ECOM spol. s. r. o., Prague, Czech Republic) at a wavelength of 480 nm. The tracking line of the separation process was recorded using an EZChrom SI software platform (Agilent Technologies, Pleasanton, CA, USA).

Fractionation of the *H. pluvialis* extract (EXT) into an astaxanthin-monoester-rich fraction (ME) and astaxanthin-diester-rich fraction (DE) was performed by CCC using a biphasic solvent system prepared by mixing *n*-heptane and acetonitrile (1:1 ratio, *v*/*v*), as previously reported [12]. The resulting mixture was vigorously shaken in a separatory funnel and allowed to stand until two immiscible liquid phases were formed. The obtained upper and lower liquid phases were separated and used as stationary and lower phases, respectively, in CCC. The sample to be processed was prepared by dissolving 5 g of microalgal extract in 10 mL of the upper phase of the selected biphasic system. To start the fractionation process, the CCC column was filled with the stationary phase (upper phase) until two column volumes had passed through. Then, column rotation was started at a speed of 800 rpm, and the mobile phase (lower phase) was pumped at a flow rate of 80 mL/min at a temperature of 28 °C. The instrument was ready for sample loading as soon as hydrodynamic equilibrium was reached in the column, which is the case when the stationary phase does not leave the column during mobile phase pumping. The retention of the stationary phase (*Sf*) in the column was estimated as previously described [12]. Shortly after ME eluted after 30 min, the elution–extrusion mode was applied to the chromatographic process to elute DE, which was strongly retained in the stationary phase. The stationary phase was extruded by switching mobile phase pumping to stationary phase pumping while the column continued to rotate [23]. The target fractions were collected manually and evaporated at 30 °C under reduced pressure, yielding 1.5 g ME and 0.1 g DE, which were subsequently analyzed using HPLC-DAD. 

### 2.4. Chromatographic Analysis

The *H. pluvialis* extract and its astaxanthin-ester-rich fractions obtained by CCC were analyzed using a Dionex UltiMate 3000 HPLC system (Thermo Scientific, Sunnyvale, CA, USA) equipped with diode array detection (DAD) and a high-resolution tandem mass spectrometry (HRMS/MS) detector with an atmospheric pressure chemical ionization (APCI) source (Impact HD mass spectrometer Bruker, Billerica, MA, USA) (HPLC-DAD-APCI-HRMS). For analysis, samples were prepared by dissolving 1 mg EXT, ME, and DE in 1 mL acetone. Sample injection volumes of 20 μL were used. Target compounds were separated using a mobile phase of water (A) and methanol (B), both containing formic acid (0.1%) to improve ionization efficiency. The mobile phase was pumped at a flow rate of 0.8 mL/min over a Luna^®^ C8 column, 100 × 4.6 mm, 3 μm (Phenomenex) at 30 °C. An elution gradient was used to pump the mobile phase according to the following pattern: 0–20 min, 20–0% A; 20–25 min, 0% A; 25–27 min, 0–20% A; 27–30 min, 20–20% A. For MS analysis, the following conditions were used: drying temperature 250 °C; drying gas flow 12 L/min; nebulizer 3 bar; spray needle voltage 4.2 kV. MS spectra were measured in the range of 50–2000 *m*/*z* in positive ionization mode with a sampling rate of 2 Hz and a collision energy of 35 eV using nitrogen as collision gas. Sodium formate clusters were used to calibrate the spectra at the beginning of each analysis. The formulas corresponding to the peaks of the molecules and fragments were calculated using Smart Formula in the Bruker Compass Data Analysis software (version 4.2). Determination of the chemical identity of the individual peaks of EXT, ME, and DE was performed by analyzing data from HPLC-APCI-HRMS in comparison with commercial standards and the literature data. 

### 2.5. Isolation and Cultivation of Mouse Splenocytes 

Experiments were performed on 8-week-old, healthy, male Balb/c mice bred at the Institute of Parasitology in its own animal facilities approved by the Ethics Committee of the State Veterinary and Food Administration of the Slovak Republic. A total of eight in vitro experiments were performed, and the spleens of three mice were aseptically isolated in each experiment. Splenocyte suspensions were obtained by gently squeezing chopped spleen tissue through 40 μm nylon filters (BD Biosciences, Darmstadt, Germany). Red blood cells were removed by incubating the cells with lysis solution (8.02% NH_4_Cl; 0.85% NaHCO_3_ and 0.37% EDTA) on ice. Splenocytes were washed twice in PBS, filtered through 40 μm nylon filters, and then resuspended in CM. Mouse splenocyte suspensions were pooled, and counted, and viability was assessed by trypan blue exclusion assay. For in vitro biological assays, stock solutions of EXT, ME, and DE were prepared at a concentration of 5 mg/100 µL DMSO. 

### 2.6. Flow Cytometric Analysis of Splenocytes 

In each experiment, the proportions of splenocyte subpopulations (lymphoid and myeloid lineages) were evaluated by flow cytometry after 24 h of incubation (3 × 10^6^/3 mL/well) in CM at 37 °C and 5% of CO_2_ in a six-well plate. Nonadherent cells were collected and counted, while adherent cells were first detached by incubation in warm Accutase solution at 37 °C for 15–20 min, followed by gentle scraping with a cell scrapper and washing with PBS. Cell samples (0.4 × 10^6^/sample) of naïve splenocytes and pooled nonadherent and adherent subpopulations after 24 h of incubation were stained with anti-mouse monoclonal antibodies to CD 45.2 (PE-Cyanine7, clone 104, eBioscience, San Diego, CA, USA), CD3 (PerCPeFluor710, clone 17A2, eBioscience, USA), CD45.R (B220, APC), and (clone RA3-6B2, eBioscience). The other samples were stained with anti-mouse antibodies against CD11c (PerCP Cyanine 5.5, clone N418, eBioscience, USA) and CD11b (FITC, clone M1/70, BioLegend, USA). Cells were stained with the antibodies for 30 min at room temperature and in the dark, washed and analyzed by flow cytometry. Phenotypic analysis was performed using a FACS Canto cell analyzer (Becton Dickinson Biosciences, Franklin Lakes, NJ, USA). The acquired data were analyzed using FACS Diva software. 

### 2.7. Trypan Blue Exclusion Test 

Splenocyte suspensions diluted to the concentration of 1 × 10^6^ cells/mL in CM, were added in duplicate to CultureSlides (Falcon Tissue Culture Treated Glass Slides, Corning, NY, USA) for each treatment, and working solutions of EXT, ME, and DE in CM were added to the cells to achieve final concentrations of 2.5, 5, 10, 20, and 40 μg/mL, respectively. Untreated splenocytes were used as controls. After 24 h of incubation, nonadherent cells (mainly lymphocytes) were collected from each well into tubes, and the number of live (white) and dead cells (blue) was counted for a total of 300 cells after the addition of trypan blue solution. The viability of cells adhering to the slide was counted in a similar manner, and the counts for nonadherent and adherent cells/well were summed. The proportions (%) of viable cells to total cells counted were calculated for the control sample and for the concentrations of EXT, ME, and DE and expressed as mean ± SD. Viability was assessed in two independent in vitro experiments. 

### 2.8. MTT Metabolic Activity Assay 

The MTT assay is commonly used to evaluate the cytotoxicity of compounds. NAD (P) H-dependent oxidoreductase enzymes reduce the tetrazolium dye 3-(4,5-dimethylthiazol-2-yl)-2,5-diphenyltetrazolium bromide to insoluble formazan crystals. In this study, MTT assay was used to evaluate the effects of astaxanthin-rich fraction, ME, and DE on the enzymatic activity corresponding to splenocyte viability. Splenocyte suspensions diluted to the concentration of 1 × 10^6^ cells/mL in CM were placed in 24-well plates (Corning, NY, USA) in triplicate for each treatment and for control. Splenocytes were treated for 24 h with EXT, ME, and DE at concentrations of 2.5, 5, 10, 20, and 40 μg/mL, and MTT (5 mg/mL in PBS) was added 4 h before the end of the assay at the concentration of 50 μL/mL. Nonadherent cell populations were transferred to microtubes and centrifuged at 956 RCF, and cell pellets containing formazan crystals were dissolved in 100 µL DMSO. Adherent cells were washed with PBS, and 100 μL DMSO was added to each well. Cell lysates from both cell compartments were then transferred to 96-well plates, and absorbance was measured at 550 nm with the reference filter at 630 nm using the Multiscan FC Plate Reader (Thermo Scientific, Finland). OD values for adherent and nonadherent cells/samples were summed and used to calculate the mean ± SD from three repeated in vitro experiments.

### 2.9. Neutral Red Uptake Assay 

The neutral red uptake assay is used to quantify cell viability and monitor the toxic effects of substances in vitro [24] and has been used to evaluate the concentration-dependent effect of EXT, ME, and DE on splenocytes. Uptake of the dye neutral red depends on the ability of cells to maintain the pH gradient through ATP production [24]; therefore, this assay is based on different physiological processes than the MTT and trypan blue uptake assays. Splenocyte suspensions (1 × 10^6^ cells/mL in CM) were placed in 24-well plates in triplicate for each treatment and control with EXT, ME, and DE at concentrations of 2.5, 5, 10, 20, and 40 μg/mL for 24 h. One hour before the end of the assay, 20 µg/10 µL of neutral red (in CM) was added to each well. Extraction of the internalized dye was performed separately for nonadherent and adherent cells using 200 µL of the extraction buffer (1% glacial acetic acid, 50% ethanol in distilled water). The optical density of the extracts from both cell counterparts/well was measured in a 96-well plate at 550 nm using a Multiscan FC Plate Reader. Subsequently, the OD values for adherent and nonadherent cells/samples were summed and used to calculate the mean ± SD from three replicate in vitro experiments.

### 2.10. Mitochondrial Membrane Potential

Mitochondria are key organelles for cell survival, and loss of mitochondrial membrane potential (Δψm) is considered a hallmark of programmed cell death (apoptosis). Splenocytes diluted at a concentration of 1 × 10^6^ cells/mL in CM were treated with 10 or 40 μg/mL EXT, ME, or DE for 24 h in 24-well plates; untreated cells were used as control. Mitochondrial potential was also evaluated in the naïve cells before incubation. To monitor the membrane potential of mitochondria, we evaluated changes in rhodamine 123 oxidation by its epifluorescence intensity. This dye is absorbed by mitochondria of living cells. Changes in dye uptake reflect Δψm and are expressed as mean fluorescence intensity (MFI). Nonadherent splenocytes were transferred to tubes, and adherent cells were detached with 300 μL of warm Accutase solution at 37 °C for 20 min, collected in tubes, and washed with CM. Then, both cell fractions/well were pooled, and rhodamine 123 solution was added at the final concentration of 10 μM. Splenocytes were incubated at 37 °C for 20 min, the supernatant was removed, and cells were resuspended in 200μL of PBS. Finally, mitochondrial membrane potential was measured in the cell suspensions after excitation of rhodamine 123 at 505 nm and emission at 535 nm. The assay was repeated three times.

### 2.11. Annexin V/Propidium Iodide Apoptosis Assay

Quantification of cells undergoing apoptosis is important to assess the effects of drugs and other substances. Splenocytes diluted at a concentration of 1 × 10^6^ cells/mL in CM were treated in 24-well plates for 24 h with 10 or 40 μg/mL of EXT, ME, or DE in triplicate; untreated cells were used as control. Naïve cells were used as intact control. Nonadherent splenocytes were transferred to tubes, and adherent cells were detached with 300 μL of warm Accutase for 20 min at 37 °C, collected into tubes, and washed. Then both cell fractions/wells were pooled, washed in cold PBS, and stained (in binding buffer) with Annexin V and propidium iodide solutions at room temperature using the BD Pharmingen Annexin V-FITC Apoptosis Detection Kit (BD Biosciences, CA, USA) according to the manufacturer’s instructions. Analysis was performed by flow cytometry using a FACS Canto flow cytometer. The proportions (%) of live cells and cells in different stages of apoptosis were evaluated using FACS Diva software. The assay was performed twice.

### 2.12. Determination of Intracellular Reactive Oxygen Species 

Mitochondria are considered the primary site of ROS production from aerobic respiration under physiological and many pathophysiological conditions. Splenocytes diluted at a concentration of 1 × 10^6^ cells/mL were treated in 24-well plates for 24 h in triplicate with 10 or 40 μg/mL of EXT. Untreated cells served as the control, and naïve cells were used as intact control. Production of intracellular ROS was determined using the fluorescent dye H_2_DCFDA, which is oxidized to the fluorescent form in the presence of ROS. Dye (5 µL/mL, 1 mM solution) was added for 4 h before the end of the assay, and generation of ROS in cells was induced by AAPH (1 mM final concentration) added one hour before end of the assay. Nonadherent splenocytes were transferred to tubes, and adherent cells were detached with 300 μL of warm Accutase for 20 min at 37 °C, collected into tubes, and washed. Then, both cell fractions/well were pooled, resuspended in 200 µL of CM, and the proportions of ROS-producing cells and MFI reflecting ROS concentration, were measured by flow cytometry using a FACS Canto flow cytometer. The assay was repeated twice.

### 2.13. Determination of Extracellular H_2_O_2_ production 

Extracellular H_2_O_2_ production was determined according to the modified method of Santos et al. [25], which is based on the oxidation of phenol red dye by H_2_O_2_ under catalytic activity of the externally added enzyme horseradish peroxidase II (HRP II). Splenocytes (1 × 10^6^ cells/mL) were diluted in HBSS without phenol red, which was added at a final concentration of 0.55 mM and was supplemented with 10 µg/mL HRP II. Cells were cultured in a 96-well plate (2 × 10^5^/well/200 µL) in triplicate for each group and treated with 10 and 40 μg/mL of EXT, ME, or DE for 2 h. The reaction was stopped by adding 10 µL of 2N NaOH. Optical density was measured at 620 nm using a Multiscan FC Plate Reader. H_2_O_2_ concentrations (in µM) were calculated using the calibration curve prepared according to the protocol described by [26]. The assay was performed twice. 

### 2.14. Detection of Nitric Oxide (NO) Production 

Splenocytes diluted to the concentration of 1 × 10^6^ cells/mL in CM were treated with 10 or 40 μg/mL astaxanthin-rich extract in 24-well plates for 24 h in duplicate. Naïve cells were used as intact control. AAPH (1 mM final concentration) was added to the cells one hour before the end of the assays to induce oxidative stress. Due to a very short half-life of NO, concentration of this molecule in the supernatants was measured as nitrite (NO_2_^−^) using the Griess reagent according to the protocol described by Ciglanová et al. [27]. Absorbance was measured at 550 nm using the Multiscan FC Plate Reader. Nitrite concentration was determined using the calibration curve with 0.1 M NaNO_3_ as the standard. The assay was performed twice.

### 2.15. Isolation of RNA from Cells and Real-Time PCR

Quantitative transcriptional profiles of genes encoding Nrf2 and housekeeping genes β-actin and GAPDH in spleen cells were determined by real-time PCR (RT-PCR). Cells (1 × 10^6^/^mL^) were plated into 24-well plates in triplicate/group and were treated with 10 and 40 μg/mL of EXT, ME, or DE. The nonadherent cells were collected in tubes, washed in PBS, and immersed in 0.5ml of TRIzol reagent (Invitrogen, Carlsbad, CA, USA). Adherent cells were then washed, immersed in 0.5 mL of TRIzol reagent, and detached with a cell scraper. Both cell fractions were pooled and used for RNA extraction. The isolated RNA was quantified using the NanoSpectrophotometer AstraGene (Harston, Cambridge, UK), and 2 µg were transcribed to cDNA using ReverseAid H Minus M-MuLV Reverse Transcriptase and oligo dT primers (Thermo Scientific, Burlington, ON, Canada). The cDNA from each sample was used as a template for quantitative PCR. RT-PCR analysis of the relative abundance of mRNA was determined using the SYBR green master mix (Sigma-Aldrich, St. Louis, MO, USA) on the BioRad CFX thermocycler (BioRad, Hercules, CA, USA). RT-PCR was performed in 20 µL reactions with detection primer pairs for nuclear factor erythroid-2-related factor 2 (Nrf2), GAPDH, superoxide dismutase (SOD1), catalase (all from [28]), and glutathione peroxidase 1 (GPx1) [29]. The primer list is shown in Table 1. 

Melting-curve analysis was performed to confirm the amplification of the single product. Ct values were normalized to housekeeping gene (GAPDH), and the relative gene expression was calculated using the 2^−∆∆Ct^ method [30] using data for naïve cells as calibration values. For the gene expression study, samples from two independent experiments were analyzed.

### 2.16. Statistical Analysis

Statistical analysis of data was performed using GraphPad Prism (version 7) for Windows (GraphPad Software, Inc., San Diego, CA, USA), and results were expressed as mean ± standard deviation calculated from triplicate samples/group of two or three independent in vitro experiments. Results were analyzed either by one-way analysis of variance (ANOVA) followed by Tukey’s post hoc test or grouped analyses using two-way ANOVA and the Sidak post hoc test. Statistically significant differences between the groups indicated by connecting lines were considered to be at least *p* < 0.05.

## 3. Results

### 3.1. Chromatographic Analysis of Carotenoid Extract and Astaxanthin Fractions

**Figure 1:**
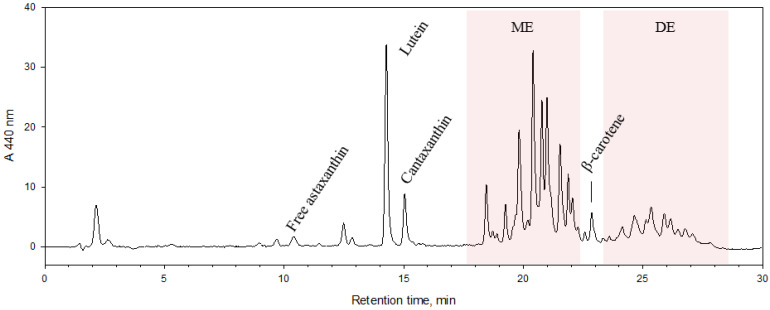
High-performance liquid chromatography with diode array detection (HPLC-DAD) chromatograms of *H. pluvialis* (EXT). The chromatogram was monitored at 480 nm.

**Figure 2:**
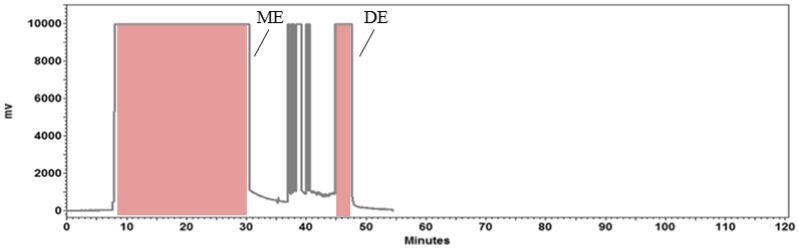
Countercurrent chromatography (CCC) method to obtain astaxanthin-monoester-rich fraction (ME) and astaxanthin-diester-rich fraction (DE) from *H. pluvialis* (EXT). Biphasic system: n-heptane and acetonitrile (1:1 ratio, *v/v*). Sample loading: 5 mg of *H. pluvialis* (EXT) dissolved in 10 mL of upper phase. Flow rate of the mobile phase: 80 mL/min. Column rotational speed: 800 rpm. Column temperature: 30 C. Detection: 480 nm.

Astaxanthin in free form, lutein, cantaxanthin, astaxanthins in the form of monoesters and diesters were identified in *H. pluvialis* extract (EXT) by HPLC-APCI-HRMS. Commercial standards of target carotenoids were used for comparison. The APCI-HRMS spectrum of peak of astaxanthin in free form showed the molecular ion [M+H]^+^ at *m*/*z* 597.3943, a fragment ion [M + H − H_2_O]^+^ at *m*/*z* 579.3844, corresponding to the cleavage of a water molecule, and a fragment ion [M+H-2H_2_O]^+^ at *m*/*z* 561.3729, formed by the loss of two water units, confirming the identity of this target compound. The identity of lutein was confirmed by its molecular ion [M + H]^+^ at *m*/*z* 569.4259 and a fragment ion [M + H − H_2_O]^+^ at *m*/*z* 551.4259. The identity of cantaxanthin was confirmed by its molecular ion [M+H]^+^ at m/z 565.4049 and a fragment ion [M + H − H_2_O]^+^ at *m*/*z* 547.3941 [31]. HPLC analysis of EXT is shown in Figure 1. ME and DE were obtained from EXT by CCC (Figure 2). The MS data of astaxanthins in ME and DE showed molecular and fragment ions corresponding to those of astaxanthin esterified with various fatty acids [31] (see Supplementary Materials, Tables S1 and S2). 

### 3.2. Characterization of the Spleen Cell Subpopulations 

**Figure 3:**
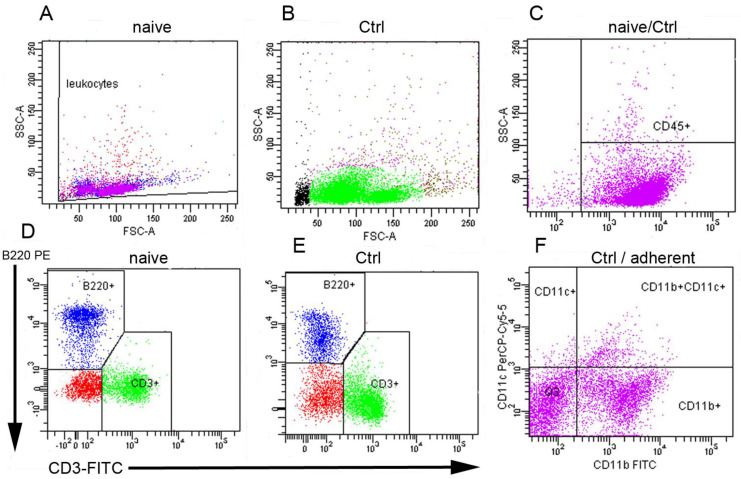
Flow cytometric analysis of spleen cells from healthy mice. Gating strategy included FSC/SSC − based distribution of naïve, freshly isolated cells (**A**), cells after 24 h of incubation (**B**), CD45+ leukocytes (**C**), proportions (%) of CD3+ T lymphocytes and B220+ B lymphocytes in naïve splenocytes (**D**) and in the control group after 24 h of incubation (**E**), and distribution of adherent myeloid cells gated on surface markers CD11c and CD11b (**F**).

Splenocytes from each mouse used in the assays were characterized by flow cytometry for the proportions of main cell phenotypes and after 24 h of incubation in the control and treated groups. The representative dot plots and gating strategy are shown in Figure 3. Lymphocytes formed the largest population, representing 81.3 ± 5% of CD45.2 + cells in naïve mice (A) and 88.2 ± 4% in cells after 24 h of culture (B). In this group, lymphocytes appeared to form two subpopulations, probably because of spontaneous activation and proliferation under standard culture conditions. In the further analysis of the lymphoid population, CD3+T cells represented 49.2 ± 4% and B220+ B lymphocytes 26.7 ± 3% in naïve cells (D) and elevated proportions of T cells (54.7 ± 3.8%) were found in the control group (E). Following cultivation, adherent cells (6.7 ± 1.2%) were gated on CD11b+, CD11c+, and CD11b+/CD11c+ myeloid-derived cells representing subpopulations of macrophages and dendritic cells. Lymphocytes accounted for the small percentage of adherent cells; therefore, in the assays, both cell fractions were collected and pooled where indicated. 

### 3.3. The Effect of H. Pluvialis Astaxanthin-Rich Extract on Viability of Splenocytes 

**Figure 4:**
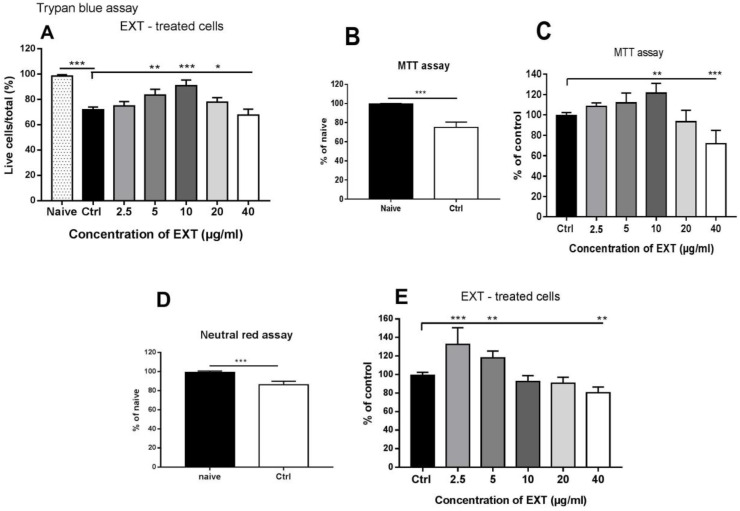
Concentration-dependent effects of astaxanthin-rich fraction (EXT) on the viability of splenocytes using trypan blue exclusion assay (**A**), MTT assay (**B**,**C**) and neutral red assay (**D**,**E**) in naïve cells and control cells after 24 h of incubation. The decrease in viable cells in the control group was calculated as the percentage of viable naïve cells (100%). The percentage of viable cells after EXT treatment was calculated as the proportion of control cells (100%). Significantly different values are indicated by * *p* < 0.0, ** *p* < 0.01, *** *p* < 0.001.

First, we investigated the concentration-dependent effect of astaxanthin-rich extract from *H. pluvialis* on splenocyte viability using three standard assays, each reflecting a different physiological process in the cells. The trypan blue exclusion assay allowed microscopic detection of the ratio of live to dead cells that passively take up dye through the cell membrane. The viability of naïve, freshly isolated splenocytes was compared with untreated cells after 24 h of incubation (Ctrl). Standard cultivation conditions with CM without reducing agent resulted in a significantly decreased number of live cells (71.90 ± 2.1, *p* < 0.001) (Figure 4A). Compared with naïve cells, the percentage of live cells cultured with EXT increased at a concentration of ≤10 µg/mL (90.98 ± 4.3%, *p* < 0.001), and viability was noticeably reduced at a concentration of 40 µg/mL (Figure 4A). 

The MTT assay allows the evaluation of the metabolic activity of the cells and is often used to measure the cytotoxic effect of bioactive compounds in vitro. Compared with naïve cells, the metabolic activity in the untreated control cells decreased to 75.21 ± 5.5% after 24 h of incubation (*p* < 0.001, Figure 4B). The effect of the astaxanthin-rich extract was determined as the percentage (%) of treated cells compared with the control (Figure 4C). Incubation with EXT had a stimulatory effect at concentrations ≤ 10 µg/mL (at 10 µg/ml, *p* < 0.05), and inhibition of metabolic activity was observed at a concentration of 40 µg/ml (*p* < 0.001). Splenocyte viability was also assessed using the neutral red uptake assay, which is based on the ability of viable cells to actively transport neutral red across membranes and, subsequently, take it up into lysosomes. Similar to what was observed in the previous assays, this cell function significantly decreased (86.01 ± 3.7%) in control cells after culturing compared with naïve splenocytes (*p* < 0.001) (Figure 4D). Exposure to EXT was stimulatory at concentrations ≤ 5 µg/mL (*p* < 0.05), and the highest concentration tested (40 µg/mL) was moderately suppressive (80.38 ± 6.1%) (Figure 4E).

### 3.4. The Effects of Astaxanthin−Rich Extract on Intracellular ROS Production 

**Figure 5:**
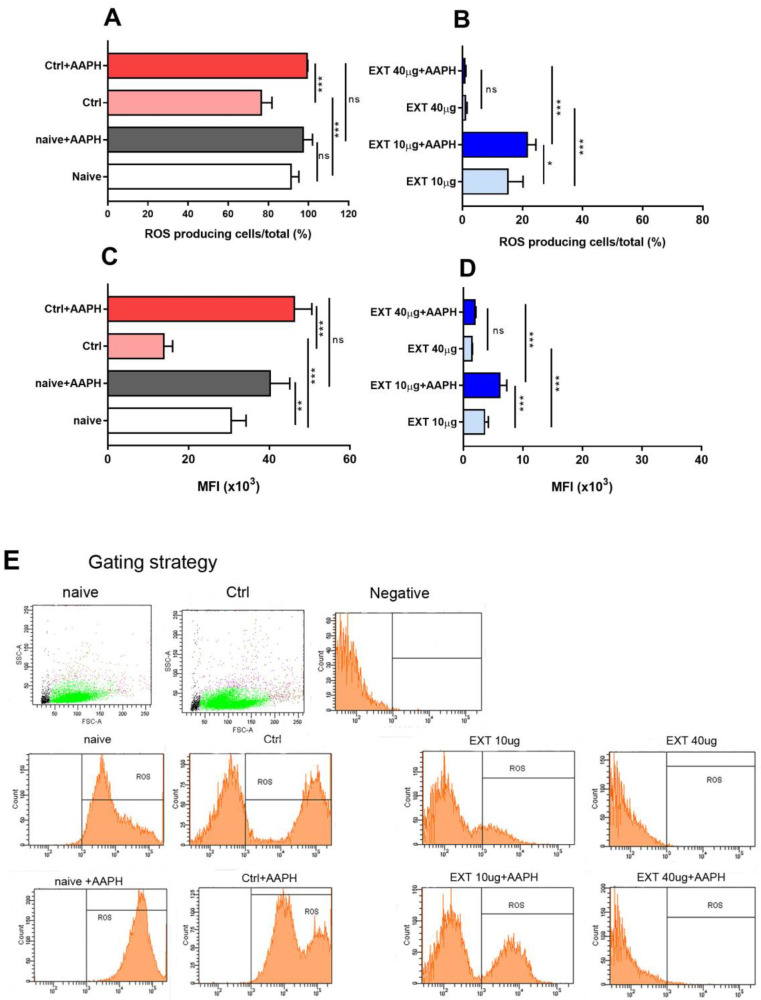
Determination of intracellular ROS in naïve spleen cells, untreated control cells, and EXT treated cells incubated for 24 h. Oxidative stress was induced by the chemical inducer 2,2′-azo-bis-(2-amidinopropane) dihydrochloride (AAPH, 1mM final concentration) added to cells 1 h before the end of the assay. (**A**,**B**), the percentage of ROS-producing cells, (**C**,**D**), MFI values for the corresponding groups, (**E**) representative histograms for indicated groups. Significantly different values are indicated by * *p* < 0.05, ** *p* < 0.01, *** *p* < 0.001.

ROS and reactive nitrogen species (RNS) are formed as natural byproducts of the activity of many cellular compartments and play an important role in cell signaling. However, increased ROS/RNS production leads to inflammation and the development of many pathological processes. In our study, intracellular ROS levels were determined using the fluorescent dye H_2_DCFDA, and AAPH was used as an oxidative stress initiator. After 24 h of cultivation, the percentage of ROS-producing cells decreased (76.56 ± 5.2, *p* < 0.001), and AAPH activated ROS production in both the naïve cells and control groups (Figure 5A). Regarding MFI, a very similar trend was observed in the naïve and control cells (Figure 5C). 

Next, we examined the ability of the ROS-scavenging capacity of astaxanthin-rich extract (EXT) at concentrations of 10 and 40 µg/mL for ROS produced by unstimulated cells and for free radicals generated by cells after addition of AAPH, which induces free radical formation by oxidation of various molecules (Figure 5B,D). In comparison with control, incubation with EXT significantly decreased the percentage of ROS-producing cells (15.20 ± 5.1% at 10 µg/mL and 1.06 ± 0.5% at 40 µg/mL). AAPH significantly increased the percentage of positive cells only at 10 µg/mL. Similarly, the mean fluorescence intensity was significantly higher at 10 µg/mL of EXT compared with the concentration of 40 µg/mL (*p* < 0.001), which nearly completely eliminated ROS generated by AAPH itself and by oxidation.

### 3.5. The Effects of Astaxanthin−Rich Extract on Extracellular Hydrogen Peroxide (H_2_O_2_) Production 

**Figure 6:**
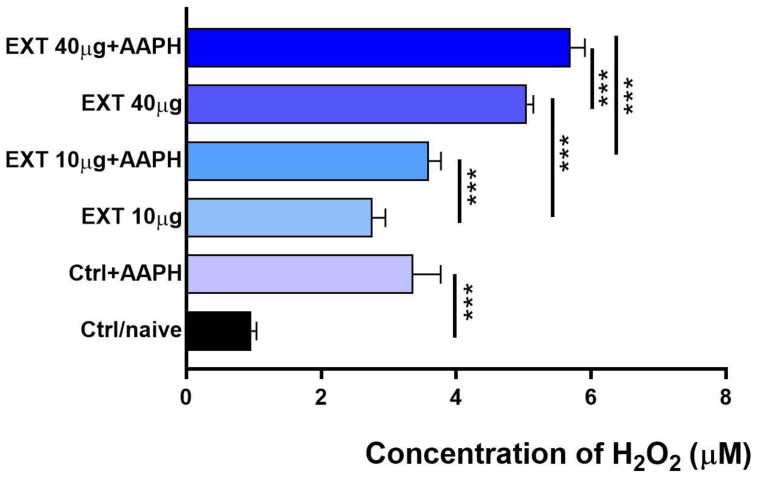
Extracellular production of hydrogen peroxide by spleen cells incubated in HBSS with phenol red for 2 h in vitro. Groups of cells were treated with EXT at the concentrations of 10 and 40 μg/mL alone or in combination with the oxidative stress inducer AAPH (1 mM final concentration). Significantly different values are indicated by *** *p* < 0.001.

Hydrogen peroxide may be involved, for example, in the process of apoptosis and signaling through the activation of caspase-3 and p53 [32]. It is produced by superoxide dismutase (SOD), which converts the highly reactive superoxide anion to the less reactive H_2_O_2_, which can easily diffuse through cell membranes. The assay was performed in HBSS solution, and cells were treated with EXT at concentrations of 10 and 40 µg/mL for 2 h (Figure 6). AAPH was used to induce oxidative stress. 

Compared to unstimulated control cells (0.95 µM ± 0.1), extracellular H_2_O_2_ production was significantly elevated in EXT-treated cells (*p* < 0.001) and more so after a concentration of 40 µg/mL (5.08 ± 0.02µM). Incubation with EXT (10 and 40 µg/mL) in combination with AAPH increased H_2_O_2_ levels only moderately compared to cells cultured with both concentrations of the extract. A stronger stimulatory effect after the addition of AAPH was observed at a concentration of 40 µg/mL of EXT.

### 3.6. The Effects of Astaxanthin−Rich Extract on Nitric Oxide Production 

**Figure 7:**
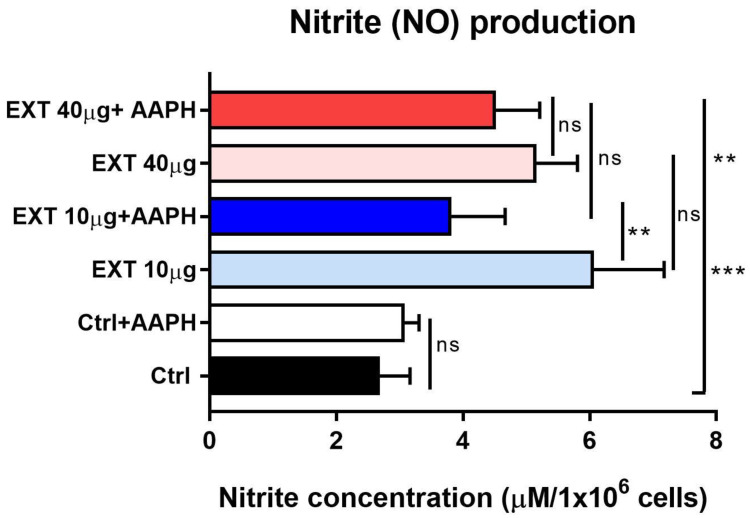
Spontaneous production of NO determined as nitrite concentration by untreated splenocytes (Ctrl) and cells treated with 10 µg/mL or 40 µg/mL of astaxanthin − rich extract (EXT) alone or in combination with AAPH. Cells were incubated with EXT for 24 h, and AAPH was added 1 h before the end of cultivation. Significantly different values are indicated by ** *p* < 0.01, *** *p* < 0.001, ns — not significant.

Nitride oxide is a diatomic free radical involved in many physiological processes including the immune response. However, reactive nitrogen species (RNS) are formed when the excess of O_2_- combines with NO and are potent oxidizing agents [33]. In the supernatants of unstimulated splenocytes cultured for 24 h, the production of NO was determined indirectly by measuring nitrite (NO_2_^-^) with the Griess reagent. Time-dependent extracellular production of NO precluded production by naïve cells. Myeloid cells, especially macrophages, are the main producers of NO. Lymphocytes, which constituted about 75–85% of spleen cells, can also produce small amounts of this molecule in healthy individuals [34], corresponding to 2.66 ± 0.55 µM in our assays. We found that EXT treatment resulted in significant higher nitrite/NO levels produced by both cell compartments within 24 h, more at concentrations of 10 µg/mL (6.04 ± 1.1 µM, *p* < 0.001) than at 40 µg/mL (5.13 ± 0.6 µM, *p* < 0.01) (Figure 7). NO is synthesized from arginine by various cells through the enzyme nitric oxide synthase. In our study, AAPH did not induce nitrite/NO synthesis and even suppressed its production following EXT treatment, similarly at both concentrations. 

### 3.7. Effect of Astaxanthin Monoesters and Diesters on Viability of Splenocytes

**Figure 8:**
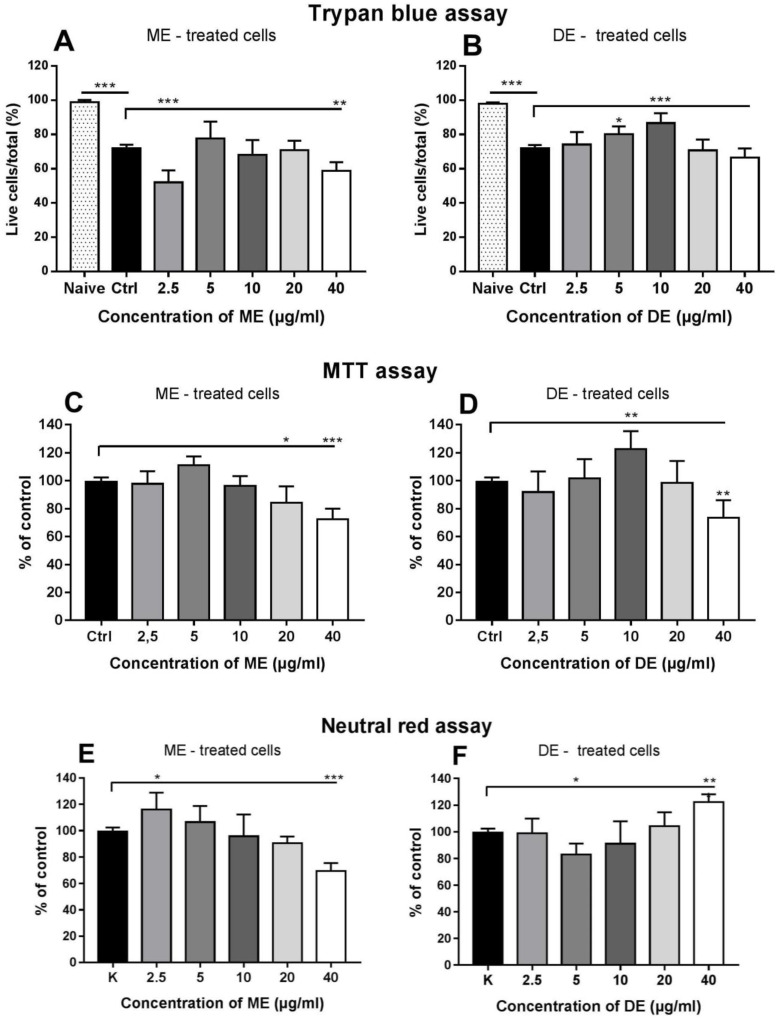
Concentration-dependent effects of astaxanthin monoesters (ME) and diesters (DE) on the viability of splenocytes using trypan blue exclusion assay (**A**,**B**), MTT assay (**C**,**D**), and neutral red assay (**E**,**F**) after 24 h of incubation. The percentage of viable cells in the treated groups was calculated as the proportion of control cells (100%). Significantly different values are indicated by * *p* < 0.0, ** *p* < 0.01, *** *p* < 0.001.

We have shown that in the carotenoid extract, most of the astaxanthin complex consists of monoesters and diesters, and only a very small proportion forms free astaxanthin. Their contribution to the final effect of EXT on splenocytes is unknown. To determine how the individual astaxanthin esters may affect cell viability as a function of concentration, we performed the same standard cytotoxicity assays as with EXT. Data are calculated as a proportion (%) of the mean for naïve cells (trypan blue assay) and as a proportion of the mean for the control group for MTT and neutral red assays. The trypan blue exclusion test showed that the low (2.5 µg/mL) and high (40 µg/mL) concentrations of ME significantly decreased the proportion of live cells, while the other concentrations showed little effect. Compared with the control group, the viability of cells treated with DE increased significantly up to a concentration ≤ 10 µg/mL (*p* < 0.001), and no cytotoxic effect was observed (Figure 8A,B). In the MTT assay, the metabolic activity of cells was significantly reduced at higher concentrations (20 and 40 µg/mL) (*p* < 0.001), and a moderate increase was observed at 5 µg/mL. Similar to the previous assay, treatment with DE stimulated cells up to a concentration of ≤10 µg/mL (*p* < 0.01), but a significant decrease in metabolic activity was observed at 40 µg/mL (*p* < 0.01) (Figure 8C,D). The uptake of neutral red by live cells is based on a different mechanism than in previous assays. In contrast to the trypan blue assay, incubation with ME at 2.5 µg/mL resulted in stimulation (*p* < 0.05), and the highest concentration tested was detrimental to this cell function. However, cells treated with DE showed a decrease in uptake at 5 µg/mL and stimulation at the highest concentration (40 µg/mL) (*p* < 0.01) (Figure 8E,F). 

### 3.8. Comparative Effects of EXT, ME, and DE on Mitochondrial Membrane Potential 

**Figure 9:**
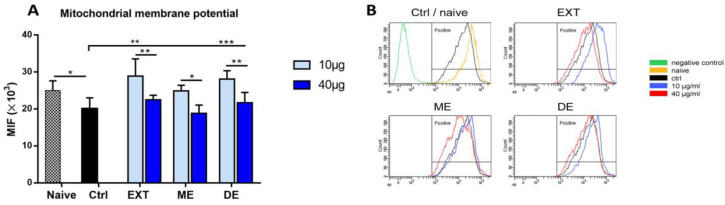
Flow cytometric analysis of mitochondrial membrane potential (MMP) expressed as mean fluorescence intensity (MFI) for rhodamine 123 in naïve splenocytes and spleen cells after 24 h of cultivation. Cells were treated with 10 µg/mL or 40 µg/mL of the extract (EXT), monoesters (ME), or diesters (DE) and changes in MFI reflect ψm levels (**A**). Representative overlay of histograms for individual cell groups (**B**). Significantly different values compared to control are indicated by * *p* < 0.0, ** *p* < 0.01, *** *p* < 0.001.

We also investigated the effect of astaxanthin EXT and AXT esters on the changes in mitochondrial dynamics by measuring changes in mitochondrial membrane potential (Δψm) by flow cytometry. The assay was performed at two concentrations (10 and 40 µg/mL) based on previous viability assays where the largest differences were observed. Data are expressed as mean fluorescence intensity (MFI) and are shown in Figure 9. In comparison with naïve cells, 24 h incubation resulted in a significant decrease in ψm (*p* < 0.05). This suppressive effect of culturing was alleviated after incubation with astaxanthin extract and DE at the concentration of 10 µg/mL (EXT, *p* < 0.01; DE, *p* < 0.001), but MMP was not affected by a concentration of 40 µg/mL. For EXT, ME, and DE, significantly lower MMPs were detected in splenocytes at this high concentration compared with a concentration of 10 µg/mL, indicating the cytoprotective effect of 10 µg/mL of EXT and its esters under standard cultivation conditions.

### 3.9. The Effect of EXT, ME, and DE on Apoptosis 

**Figure 10:**
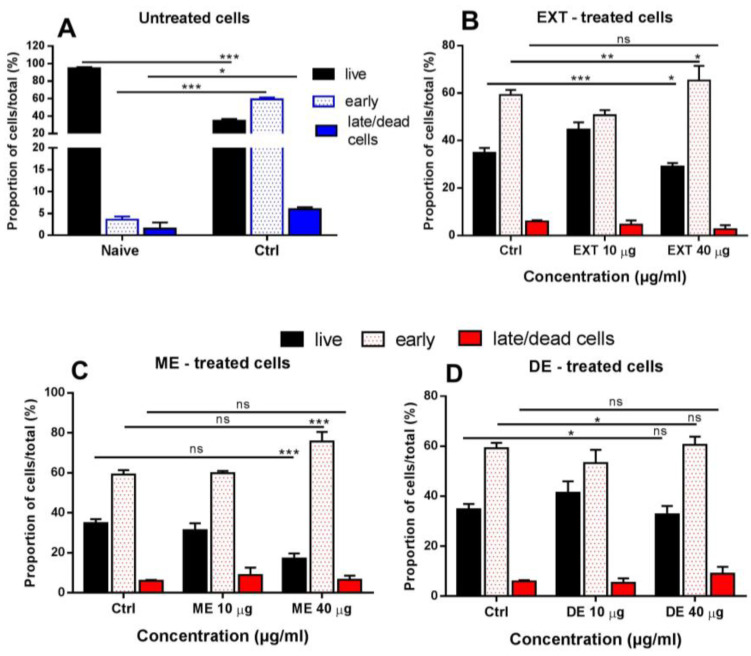
The effect of cultivation of untreated cells (**A**), astaxanthin-rich extract (EXT) (**B**), monoesters (ME) (**C**), and diesters (DE) (**D**) used at the concentrations of 10 µg/mL or 40 µg/mL on apoptosis in splenocytes. The proportions of live cells, cells in the early stage of apoptosis, and in the late phase/dead cells were determined following staining with Annexin V and propidium iodide in the spleen cells after 24 h culture. Significantly different values between groups are indicated by connecting lines and shown as * *p* < 0.05, ** *p* < 0.01, *** *p* < 0.001, ns — not significant.

Apoptosis is one of the best characterized mechanisms involved in the physiology and, also, in the pathology of eukaryotic organisms. An excess of ROS is one of the key stimuli in of the intrinsic pathway in the induction of cell death. Next, we evaluated the effects of EXT, ME, and DE at the concentrations of 10 µg/mL and 40 µg/mL on the percentage of live cells, cells in the early stage of apoptosis, and dead/late apoptotic cells by flow cytometry. In parallel, we compared naïve cells and cells after 24 h incubation under standard conditions in CM (Ctrl group). The majority of freshly isolated splenocytes were alive (94.81 ± 1.2%), and only 3.61 ± 0.7% were in the early stage. However, after 24 h of incubation, only 34.80 ± 2.0% were still alive, and 59.22 ± 2.5% of the cells were in the early stage of apoptosis (Figure 10A). Incubation with 10 µg/mL EXT partially prevented the induction of changes leading to apoptosis, resulting in a significantly higher proportion of live cells (44.65 ± 3.0%, *p* < 0.001) (Figure 10B), which correlates with the increase in mitochondrial membrane potential. 

In comparison with the control, the higher concentration of EXT (40 µg/mL) proved to be less favorable for cell survival, and 65.32 ± 6.1% of the cells were in the early stage. The effect of monoesters (ME) at 10 µg/mL did not significantly modulate the apoptotic process; however, the high concentration was pro-apoptotic, as indicated by an increased population of cells in the early stage of apoptosis (75.65 ± 4.3%) and a decreased proportion of living cells (17.10 ± 2.5%, *p* < 0.001) (Figure 10C). In contrast, this high concentration of DE was not harmful to cells, and a lower concentration (10 µg/mL) resulted in a significantly higher percentage of live cells (41.42 ± 4.5%) and a decreased number of early stage apoptotic cells (53.30 ± 5.2%, *p* < 0.05) (Figure 10D). 

### 3.10. Modulation of Gene Expression for ROS-Controlling Enzymes with EXT, ME, and DE 

**Figure 11:**
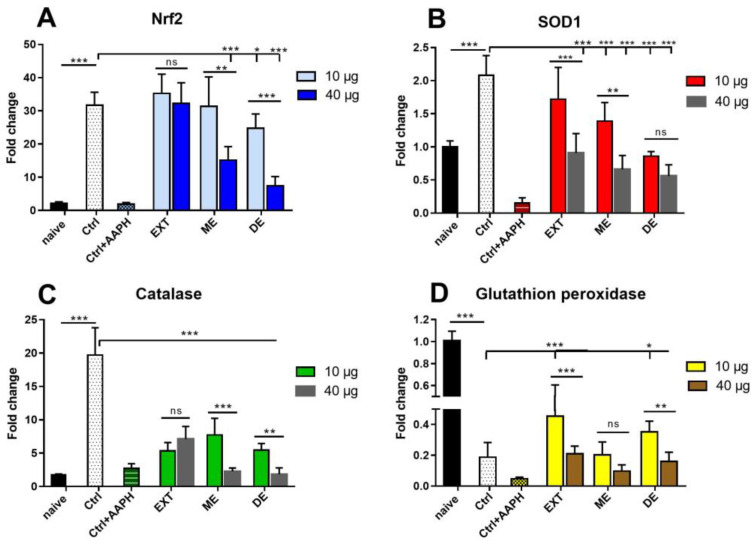
Effect of astaxanthin − rich extract (EXT), monoesters, and diesters on mRNA levels of redox-controlling enzymes Nrf2 (**A**), superoxide dismutase 1 (SOD1) (**B**), catalase (**C**), and glutathione peroxidase 1 (GPx1) (**D**) in naïve, control, and treated splenocytes. AAPH was added to the control group 1 h before the end of cultivation. Cells were treated with 10 or 40 µg/mL of EXT, ME, and DE for 24 h and untreated cells served as control. Naïve freshly isolated splenocytes were used as intact control for cultivation conditions. Significantly different values are indicated by * *p* < 0.05, ** *p* < 0.01, *** *p* < 0.001, ns—not significant.

To determine whether astaxanthin and its monoesters and diesters can affect enzymes involved in maintaining redox balance in cells under standard culture conditions, the gene expression study was performed with concentrations of 10 and 40 µg/mL compounds. In the calculation of relative gene expression, the data obtained in freshly isolated naïve splenocytes were used as a calibration value. We evaluated the expression level of the transcription factor Nrf2, which is involved in the protection of the organism from oxidative stress, and the enzymes superoxide dismutase 1 (SOD1), as well as catalase and glutathione peroxidase 1, which are both endogenous antioxidant enzymes catalyzing reduction in H_2_O_2_. The mRNA levels of Nrf2, SOD1, and catalase were significantly upregulated within 24 h of cultivation (*p* < 0.001), suggesting that cells undergo physiological changes that lead to oxidative stress. Surprisingly, expression of GPx1 declined after culturing, and addition of AAPH nearly completely abolished transcription of all three genes. While cultivation with 10 µg/mL of EXT and ME did not alter the expression of Nrf2, the expression of this gene was significantly downregulated by the treatment with DE at both concentrations (Figure 11A). 

Superoxide dismutase 1 (SOD1) converts O_2_^−^ to H_2_O_2_, and its gene expression was significantly reduced in treated cells compared with control, most strongly after incubation with DE (*p* < 0.001) (Figure 11B). The concentration of 40 µg/mL was more suppressive than a concentration of 10 µg/mL for all three compounds. The enzyme catalase converts H_2_O_2_ to H_2_O and molecular O_2,_ and higher expression levels were observed than for SOD1. The mRNA transcripts for this enzyme were downregulated by all three compounds (*p* < 0.001). A similar effect was observed for EXT, ME, and DE at lower concentrations, whereas higher concentrations of ME and DE significantly suppressed gene expression for catalase (Figure 11C). In contrast with high mRNA levels for catalase, very low expression for GPx1 was observed after 24 h cultivation in splenocytes. A decrease in GPx1 expression in the control group was partially restored after treatment with 10 µg/mL of EXT and DE (Figure 11D). 

## 4. Discussion 

In this study, CCC was shown to be efficient for the recovery of the astaxanthin-monoester-rich fraction (ME) and astaxanthin-diester-rich fraction (DE) from *H. pluvialis* extract (EXT). In CCC, solvent systems with a wide range of polarities can be used, allowing efficient isolation of compounds with great chemical diversity. Furthermore, since the stationary phase in CCC is liquid, it is possible to inject a large amount of extract and consequently obtain large amounts of fractions/compounds [12,23]. These advantages are particularly important for bioassays and for chemical identification. The CCC platform used in this study served as a chromatographic preparative tool to support bioassays. It is clear that further research is needed to develop more selective CCC methods aimed at isolating the individual astaxanthin monoesters and diesters and the other carotenoids present in EXT to determine their contribution to the observed effect of EXT and for potential commercial application. According to [35], astaxanthin esters had higher thermal stability and higher bioavailability than astaxanthin in free form. However, our study is limited only to the in vitro context without metabolic implications but is of great importance in determining their biological potential.

Mouse spleen cells were used to study the concentration-dependent biological and antioxidant effects of astaxanthin-rich extract, its monoesters, and diesters. To clarify whether cultivation in complete RPMI medium and standard conditions (5% CO_2_, humidity, and access to atmospheric oxygen ~18–20%) can influence cell physiology, naïve, freshly isolated splenocytes were compared with untreated cells after 24 h of culture. Surprisingly, a significant decline was observed in almost all parameters, suggesting that standard cultivation conditions altered cell physiology. In recent years, many reports have shown that standard cultivation conditions for different cells do not reflect physiological conditions in vivo, where organs and tissues are characterized by their own unique “tissue normoxia” or physioxia status [36,37]. It appears that the values corresponding to the physioxia range from 11% to 1% O_2,_ whereas current in vitro experiments on mammalian cell cultures are usually performed at atmospheric O_2_ levels (18–19.9%) [37]. In the spleen of rats, oxygen tension was approximately 10 ± 2.4% [38]. Thus, cells cultured under standard conditions are actually exposed to elevated oxygen levels (hyperoxia), which significantly affect ROS production [39], mitochondrial functions [40], and drug response [41]. 

We found that T and B lymphocytes constituted approximately 80–90% of the splenocyte population, and spontaneous proliferation started after culturing under standard conditions, as shown by flow cytometry and our previous observations. The most significant metabolic change after activation of naïve T-cells, which use oxidative phosphorylation for ATP production, is an increase in glucose metabolism and aerobic glycolysis, a specific metabolic adaptation known as the Warburg effect [42,43,44]. Splenocyte viability was assessed using three standard assays, which are based on different physiological processes in the cells. The trypan blue exclusion assay is the most common and widely used method for determining cell viability, and the dye passively permeates the cell membrane of dying cells [45]. In the MTT assay, the reduction of this dye to insoluble crystal-forming formazan in cells is usually attributed to nicotinamide adenine dinucleotides (NADH) and dehydrogenases associated with the endoplasmic reticulum rather than primarily by the mitochondrial succinate [46,47]. The neutral red uptake assay is based on the ability of viable cells to take up the supravital dye neutral red by pinocytosis and bind it in lysosomes [24]. In all three assays, the proportion of live cells significantly decreased (72–86%) after 24 h incubation, indicating that the cells were exposed to nonphysiological conditions, likely hyperoxia. Similarly, [40] demonstrated that super-physiological O_2_ levels significantly reduced important cellular activities and signaling in several cell lines compared to physiological levels in cell culture (18% versus 5%). 

In trypan blue and MTT assays, co-culture with concentrations of EXT up to 10 µg/mL prevented a significant decrease in cell viability; however, transport of the neutral red dye across membranes was inhibited at concentrations up to 5 µg/mL. In contrast, a decrease in cell viability was observed at a concentration of 40 µg/mL in all three assays. Immune cells are particularly sensitive to oxidative stress because of a high content of polyunsaturated fatty acids in their plasma membranes [6]. Therefore, the astaxanthin-rich extract probably prevented cell membrane damage by protecting their components from oxidation. The extract studied contained approximately 95% esterified forms of astaxanthin isolated as the monoester fraction (~70%) and the diester fraction (~25%), so we compared their concentration-dependent effects on cell viability. We found some differences between the three assays and, while ME moderately affected viability up to a concentration of 20 µg/mL, DE prevented a significant decrease in viable cells up to a concentration of 10 µg/mL. In addition to the increase in neutral red uptake after incubation with 40 µg/mL, these concentrations of ME and DE did not show a cytoprotective effect. We assume that this was due to the complete removal of ROS, which are required at physiological concentrations for normal cellular metabolism and signaling. Numerous studies have shown that reactive oxygen species act as important physiological regulators of intracellular signaling pathways, activating or deactivating various receptors, proteins, and ions [48,49,50,51]. Esterification increases the solubility of carotenoids in the lipids with which they are naturally associated, thus increasing their bioavailability [52], which may explain the cytoprotective activity of astaxanthin diesters at lower concentrations. It appears that in astaxanthin-rich extract, the bioactivities of both esters are balanced. Using several cell lines, [53] showed that synthetically prepared carotenoid samples containing the highest levels of AXT monoesters (~56%) had stronger antioxidant and anti-inflammatory activities than samples containing more AXT diesters and non-esterified AXT, which is very similar to composition of the natural mixture found in EXT.

Changes in mitochondrial dynamics were assessed by evaluating changes in mitochondrial membrane potential (MMP) (Δψm) using the fluorescent dye rhodamine 123 [54,55]. In addition, the effect of cultivation and tested compounds on apoptosis was evaluated, as mitochondrial dysfunction and loss of mitochondrial membrane potential are associated with the intrinsic apoptosis pathway [56]. Compared with naïve cells, MMP in splenocytes decreased after 24 h of cultivation, which was associated with the transition of approximately 55% of cells to the early stage and approximately 5% to the late phase of apoptosis. A high number of control cells in the early stage of apoptosis proved that cell culture conditions adversely affected cell viability, confirming the results of our previous tests. It has been shown that even short-term hyperoxia exposure leads to mitochondrial dysfunction, which is manifested by a decrease in membrane potential [57,58]. To see whether EXT, ME, and DE can restore mitochondrial functions, we examined their effects at the concentrations of 10 and 40 μg/mL, which showed either positive or negative effects, resp., in viability assays. Compared with the control group, incubation of cells with 10 μg/mL of EXT, ME, and DE for 24 h increased MMP to levels found in naïve splenocytes; however, the highest concentration tested was not effective in protecting cells, as evidenced by reduced MMP. Similarly, 10 μg/mL of EXT and DE increased the proportions of live cells and decreased the percentage of apoptotic cells compared with the control, whereas ME was not effective. In agreement with previous results, the higher concentration significantly increased the proportion of cells in the early stage of apoptosis. The functionality of carotenoids is determined by their subcellular localization, and mitochondria of lymphocytes contributed to their significant uptake [6]. It seems that the very potent antioxidant capacity of astaxanthin and its esters, especially at high concentrations, abolished the normal physiological production of the ROS in splenocytes required for cell signaling, which probably contributed to the transition of cells to the early apoptotic stage.

To further investigate the antioxidant capacity of the extract containing both esterified forms of astaxanthin, oxidative stress in splenocytes was chemically induced by exposing them to AAPH, in addition to hyperoxia in culture. More than 90% of naïve cells retained the ability to produce physiological levels of ROS, and their percentage decreased significantly after cultivation (~70%). However, the production of ROS in control cells induced by AAPH was preserved, indicating that hyperoxia in culture probably induces lymphocytes hyporesponsiveness in agreement with the decrease in MMP. A direct correlation between the level of T lymphocyte hyporesponsiveness and the concentration of ROS in culture was found by [20]. The ability of cells to produce ROS was partially reduced by low concentrations of EXT and was almost completely eliminated at 40 μg/mL of EXT, even after addition of AAPH. The same trend was observed for MFI values, demonstrating a very strong scavenging ability of the naturally occurring mixture of esterified forms of AXT. It is plausible that these compounds also interfere with mitochondrial and possibly other ROS-producing enzymes and transcription factors that regulate ROS levels. 

In addition to mitochondria, the superoxide anion O_2_^-^ and the hydrogen peroxide H_2_O_2_ (ROS) are also formed in the endoplasmic reticulum, peroxisomes, and the cytosol by enzymes such as respiratory complexes, NADPH oxidases (NOX), xanthine oxidase, and others [37]. Moreover, NAPPH oxidase 4 primarily produces H_2_O_2_ rather than superoxide, and its levels are influenced by the O_2_ content in the culture [39]. The superoxide anion can be rapidly converted to H_2_O_2_ by the antioxidant enzyme superoxide dismutase 1 (SOD1), maintaining a low superoxide equilibrium state [59,60]. Subsequently, the excess of H_2_O_2_ is converted to water and molecular oxygen O_2_ by the antioxidant enzyme catalase [61,62] and glutathione peroxidase 1 [63], which is present in almost all aerobic organisms. H_2_O_2_ production by splenocytes was determined after a two-hour incubation in HBSS with the redox-sensitive molecule phenol red [25], which did not allow comparison between naïve and control cultures. Extracellular H_2_O_2_ concentration was significantly higher at 40 μg/mL than at 10 μg/mL and was proportionally increased in cultures treated with AAPH together with EXT. Since AXT and its esters, unlike other carotenoids, do not exhibit prooxidant activity, we assume that AXT esters present in EXT stimulate, proportionally to their concentration, the conversion of ROS to H_2_O_2_, which is considered a weak oxidant compared to O_2_^-^ and is an important signaling molecule [1]. 

In contrast to the direct oxygen-sensing mechanisms in hypoxia, the cellular response to hyperoxia is regulated by molecular mechanisms sensitive to H_2_O_2_ [58], the most important of which is the pathway regulated by Nrf2. Nrf2 is ubiquitously and constitutively expressed by cells, providing a prompt protective response to oxidative, inflammatory, and metabolic stresses [64,65]. We also examined the expression of genes related to antioxidant activities to see how the AXT-rich extract and its esters modulate mRNA transcription levels for Nrf2, SOD1, catalase, and glutathione peroxidase (GPx1) in splenocytes exposed to hyperoxia-like culture. Compared with naïve cells, mRNA transcription of all genes, except of GPx1, was upregulated in the control group. Indeed, upregulation of Nrf2 has been demonstrated in severe hyperoxia (> 60% O_2_) in various biological systems [58]. Surprisingly, adding AAPH almost completely abolished gene transcription, although maximal stimulation was expected at least for Nrf2. This might be related to the detrimental effect of the AAPH molecule on this biological process. Therefore, we did not further use this chemical stress inductor in treated splenocyte cultures. While at both concentrations of EXT (10 and 40 µg/mL), Nrf2 gene transcription remained upregulated, incubation with ME and intense DE resulted in a significant decrease, and, as previous results showed, the high concentration was more suppressive. A very similar trend in mRNA transcription was observed for SOD1 and catalase, which was more highly expressed than SOD1 and GPx1, suggesting that catalase had the main role in H_2_O_2_ reduction. 

Carotenoids, including AXT, are cytosolically soluble and electrophilic compounds and, therefore, are able to stimulate the translocation of Nrf2 to the nucleus and subsequently stimulate the expression of antioxidant enzymes such as SOD and catalase, leading to reduced lipid peroxidation and oxidative stress [50,66,67]. Xanthophylls, which include astaxanthin and its esters, are also more electrophilic compared to other carotenoids, making them more bioactive and, thus, more stimulatory of transcription factor expression [67]. We assume that all three AXT preparations, depending on their concentration in cell culture, in addition to direct scavenging activity, convert excess of ROS to H_2_O_2_, which was confirmed by a strong reduction in intracellular ROS in cells. Their levels (MFI) directly correlate with SOD1 expression after treatment with EXT, suggesting that mRNA levels are controlled by superoxide levels. We demonstrated that the individual esters lowered SOD1 mRNA levels even more; however, their different proportions in EXT must be taken into account when comparing the effect of EXT with that of ME and DE. These data imply a stronger ROS-scavenging activity of DE, which may be supported by the higher percentage of live cells and the higher MMP at the same concentration. Splenocytes cultured for a short time (2 h) in a different cultivation system (HBBS with phenol red) produced very low H_2_O_2_ concentrations, indicating that cells probably have not yet responded to the conditions associated with hyperoxia by activating Nrf2 and SOD1. Increased H_2_O_2_ concentrations after incubation with EXT and AAPH appear to be the result of Nrf2 and SOD1 stimulation leading to rapid conversion of ROS to H_2_O_2_. Compared with control cells, catalase mRNA transcription was downregulated, and GPx1 was moderately upregulated, and we hypothesize that EXT, ME, and DE were able to eliminate H_2_O_2_ excess to physiological levels during this period. As the activation of antioxidant enzymes is sensitive to the concentration of ROS/H_2_O_2_, this could explain the decreased gene expression for these enzymes. 

Nitric oxide is an important lipid-permeable signaling molecule and is produced by inducible nitric oxide synthase (iNOS) in immune cells, particularly macrophages and NK cells, in response to various inflammatory mediators [68,69]. NO synthesis has also been detected in T lymphocytes in response to chemokines and cytokines at low levels [70]. We nondirectly determined the spontaneous extracellular production of NO as nitrite by spleen cells within 24 h of cultivation under hyperoxia-like conditions and the effects of EXT at concentrations of 10 and 40 µg/mL. Compared with the control, the NO concentration was significantly increased in the presence of EXT and more so at lower concentrations, whereas AAPH did not further increase NO. Sun et al. [71] showed that ROS and RNS regulate iNOS function by shifting the balance of O_2_^-^ and NO production and that superoxide anion can greatly reduce NO generation and directly inactivate NO [72]. Consistent with these reports, in our study, elevated NO levels produced by lymphocytes after EXT treatment correlate with significantly decreased oxidative stress and ROS concentration, supporting our previous results. 

The design of this study is based on an in vitro model with the aim of determining the biological potential of astaxanthin fractions, paving the way for further in vivo studies. They would allow us to measure the pharmacokinetic and pharmacodynamic parameters, which is not the case in our study. 

## 5. Conclusions 

Overall, the results of our study showed strong cytoprotective effects on mouse spleen cells by astaxanthin-rich carotenoid extract (EXT), astaxanthin-monoester-rich fraction (ME), and astaxanthin-diester-rich fraction (DE) obtained from the microalgae *Haematococcus pluvialis*. The decrease in viability and pro-apoptotic changes observed in unstimulated splenocytes during culture in standard RPMI medium and conditions were restored or stimulated after co-cultivation with lower tested concentrations up to 10 µg/mL in the order EXT > DE > ME. Higher concentrations of the tested carotenoid extract were showed to have moderate adverse effects. It appears that the access of atmospheric oxygen (~18–20%) during incubation, referred to as hyperoxia, led to a whole range of negative consequences for cell physiology. The protective mechanism was primarily associated with the concentration-dependent scavenging activity for ROS, in parallel with their conversion to H_2_O_2_ and eventually to oxygen and water/alcohol. Consequently, hyperoxia-induced activation of the antioxidant genes Nrf2, SOD1, catalase, and GPx1 in cell cultures was attenuated after treatment with EXT, DE, and ME in correlation with ROS/RNS levels and was consistent with cell viability. These results suggest that AXT and its esters, present in vivo at physiologically achievable concentrations, have the ability to precisely regulate the levels of ROS/RNS by direct action and also by modulating the cellular signaling pathways necessary for proper cell functioning.

## Figures and Tables

**Table 1:** List of oligonucleotides and their sequences.

Gene	Orientation	Sequence
Nrf2	forward	5′-CTTTAGTCAGCGACAGAAGGAC-3′
	reverse	5′-AGGCATCTTGTTTGGGAATGTG-3′
GAPDH	forward	5′-AGGTCGGTGTGAACGGATTTG-3′
	reverse	5′-TGTAGACCATGTAGTTGAGGTCA-3′
Superoxide dismutase 1 (SOD1)	forward	5′-AACCAGTTGTGTTGTCAGGAC-3′
	reverse	5′-CCACCATGTTTCTTAGAGTGAGG-3′
Catalase (CAT)	forward	5′-AGCGACCAGATGAAGCAGTG-3
	reverse	5′-TCCGCTCTCTGTCAAAGTGTG-3′
Glutathion peroxidase 1 (GPx1)	forward	5′-CTCACCCGCTCTTTACCTTCCT-3′
	reverse	5′-ACACCGGAGACCAAATGATGTACT-3′

## Data Availability

Data are contained within the article.

## Supplementary Materials

The following supporting information can be downloaded at https://www.mdpi.com/article/10.3390/antiox12061144/s1; Table S1: Mass spectrometry (MS) data of astaxanthin monoesters present in astaxanthin monoesters-rich fraction ME; Table S2: Mass spectrometry (MS) data of astaxanthin diesters present in astaxanthin diesters-rich fraction (DE).
