# Supplementary material for: Astaxanthin Extract from Haematococcus pluvialis and Its Fractions of Astaxanthin Mono- and Diesters Obtained by CCC Show Differential Antioxidant and Cytoprotective Effects on Naïve-Mouse Spleen Cells
Source: Antioxidants (Basel). 2023 May 24;12(6):1144. doi: 10.3390/antiox12061144 (PMC10295246; doi:10.3390/antiox12061144)
Supplement: Supplementary file 1 [file antioxidants-12-01144-s001.zip › antioxidants-2338081-supplementary.pdf]

Supplementary Table 1. Mass spectrometry (MS) data of astaxanthin monoesters present in astaxanthin monoesters-rich fraction ME.

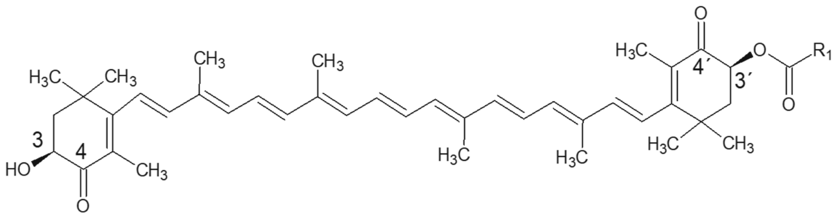

t_R [min]	λ_{max} [nm]	Molecular ion (*m*/*z*) $[M+H]^+$	Fragment ion (*m*/*z*) $[M+H - FA]^+$	Fragment lost (*m*/*z*) (FA)	Astaxanthin monoesters
17,5	-	827,5659	579,3868	248,1791	AXT-FA(16:4)
18	-	829,5696	579,3797	250,1899	AXT-FA (16:3)
18,4	476	855,5923	579,3847	276,2076	AXT-FA (18:4)
18,6	469	831,5943	579,384	252,2103	AXT-FA (16:2)
18,9	474	857,6075	579,3832	278,2243	AXT-FA (18:3)
18,9	476	881,6038	579,3838	302,2200	AXT-FA (20:5)
19	476	807,5935	579,3845	228,2090	AXT-FA (14:0)
19,1	466	845,6082	579,3836	266,2246	AXT-FA (17:2)
19,3	471	833,6098	579,384	254,2258	AXT-FA (16:1)
19,3	471	883,6242	579,3854	304,2388	AXT-FA (20:4)
19,6	476	859,6256	579,3855	280,2401	AXT-FA (18:2)
19,8	467	847,6165	579,3867	268,2298	AXT-FA (17:1)
19,9	475	835,6246	579,3849	256,2397	AXT-FA (16:0)
19,9	476	885,6415	579,3853	306,2562	AXT-FA (20:3)
20	474	835,6247	579,385	256,2397	AXT-FA (16:0)
20,2	475	861,6411	579,3855	282,2556	AXT-FA (18:1)
20,4	467	887,6534	579,3846	308,2688	AXT-FA (20:2)
20,8	473	863,6560	579,3848	284,2712	AXT-FA (18:0)
21,5	474	891,6875	579,3855	312,3020	AXT-FA (20:0)

AXT: astaxanthin. FA: Fatty acid. t_R: retention time. C16:4=hexadecatetraenoic acid. C16:3=hexadecatrienoic acid. C18:4=octadecatetraenoic acid. C16:2=hexadecadienoic acid. C18:3=linolenic acid. C20:5=eicosapentaenoic acid. C14:0=myristic acid. C17:2=heptadecadienoic acid. C16:1=palmitoleic acid. C20:4=arachidonic acid. C18:2=linoleic acid. C17:1=heptadecenoic acid. C16:0=palmitic acid. C20:3=dihomo-γ-linolenic acid. C18:1=oleic acid. C20:2=eicosadienoic acid. C18:0=stearic acid. C20:0=arachidic acid.

Supplementary Table 2. Mass spectrometry (MS) data of astaxanthin diesters present in astaxanthin diesters-rich fraction (DE).

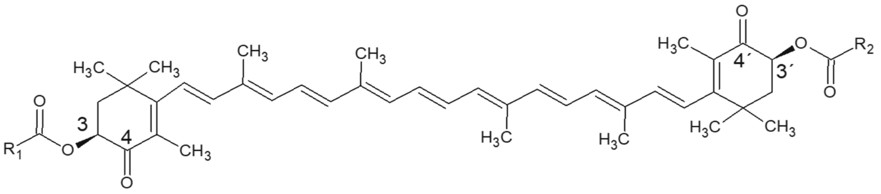

t_R [min]	Molecular ion (*m*/*z*) [M+H]$^+$	Astaxanthin diesters
23,4	1117,8257	AXT-FA/FA(18:3/18:3)
23,9	1119,8385	AXT-FA/FA (18:3/18:2)
23,9	1069,8207	AXT-FA/FA (18:2/14:0)
24,1	1095,8399	AXT-FA/FA (18:3/16:0)
24,2	1145,8529	AXT-FA/FA (20:4/18:2)
24,3	1121,8537	AXT-FA/FA (18:2/18:2)
24,5	1071,8410	AXT-FA/FA (18:1/14:0)
24,6	1097,8548	AXT-FA/FA (18:2/16:0)
24,9	1123,8716	AXT-FA/FA (18:2/18:1)
25,1	1073,8544	AXT-FA/FA (16:0/16:0)
25,2	1149,8855	AXT-FA/FA (18:1/20:3)
25,3	1099,8712	AXT-FA/FA (18:1/16:0)
25,5	1125,8841	AXT-FA/FA (18:1/18:1)
26,0	1101,8872	AXT-FA/FA (16:0/18:0)
26,2	1128,9021	AXT-FA/FA (18:0/18:1)
26,5	1153,9160	AXT-FA/FA (20:0/18:2)
27	1129,9201	AXT-FA/FA (18:0/18:0)

AXT: astaxanthin. FA: Fatty acid. t_R: retention time. C18:3=linolenic acid. C18:2=linoleic acid. C14:0=myristic acid. C16:0=palmitic acid. C20:4=arachidonic acid. C18:1=oleic acid. C20:3=dihomo-γ-linolenic acid. C18:0=stearic acid. C20:0=arachidic acid.
